# Essential Role of Domain III of Nonstructural Protein 5A for Hepatitis C Virus Infectious Particle Assembly

**DOI:** 10.1371/journal.ppat.1000035

**Published:** 2008-03-28

**Authors:** Nicole Appel, Margarita Zayas, Sven Miller, Jacomine Krijnse-Locker, Torsten Schaller, Peter Friebe, Stephanie Kallis, Ulrike Engel, Ralf Bartenschlager

**Affiliations:** Department of Molecular Virology, University of Heidelberg, Heidelberg, Germany; University of Lausanne, Switzerland

## Abstract

Persistent infection with the hepatitis C virus (HCV) is a major risk factor for the development of liver cirrhosis and hepatocellular carcinoma. With an estimated about 3% of the world population infected with this virus, the lack of a prophylactic vaccine and a selective therapy, chronic hepatitis C currently is a main indication for liver transplantation. The establishment of cell-based replication and virus production systems has led to first insights into the functions of HCV proteins. However, the role of nonstructural protein 5A (NS5A) in the viral replication cycle is so far not known. NS5A is a membrane-associated RNA-binding protein assumed to be involved in HCV RNA replication. Its numerous interactions with the host cell suggest that NS5A is also an important determinant for pathogenesis and persistence. In this study we show that NS5A is a key factor for the assembly of infectious HCV particles. We specifically identify the C-terminal domain III as the primary determinant in NS5A for particle formation. We show that both core and NS5A colocalize on the surface of lipid droplets, a proposed site for HCV particle assembly. Deletions in domain III of NS5A disrupting this colocalization abrogate infectious particle formation and lead to an enhanced accumulation of core protein on the surface of lipid droplets. Finally, we show that mutations in NS5A causing an assembly defect can be rescued by trans-complementation. These data provide novel insights into the production of infectious HCV and identify NS5A as a major determinant for HCV assembly. Since domain III of NS5A is one of the most variable regions in the HCV genome, the results suggest that viral isolates may differ in their level of virion production and thus in their level of fitness and pathogenesis.

## Author Summary

The hepatitis C virus (HCV) is a major cause of acute and chronic liver diseases worldwide. In spite of high medical need there is no selective antiviral therapy available and a prophylactic vaccine is not in sight. Their development requires cellular replication systems that have become available just recently. One of the most fascinating insights gained with these systems is the finding that infectious HCV particles assemble in close association with an intracellular lipid storage compartment termed lipid droplets. In this study we show that nonstructural protein 5A (NS5A), a component of the viral RNA replication machinery is a key factor for the formation of infectious HCV particles. We identify a distinct domain in NS5A as the primary “assembly determinant” and show that NS5A and the core protein, which is a major constituent of the virus particle, accumulate on the surface of lipid droplets. Deletions in NS5A disrupting the colocalization of core and NS5A on lipid droplets abrogate infectious HCV production. These studies unravel a unique pathway of infectious virus formation and identify NS5A as a factor modulating HCV replication and assembly and thus viral fitness.

## Introduction

The hepatitis C virus (HCV) is a major causative agent of acute and chronic liver diseases worldwide [1]. A hallmark of HCV infection is the high persistence, which is unusual for a virus of this group and which has been explained by numerous active and passive immune evasion strategies [2],[3]. Although primary infection is often asymptomatic or associated with mild and non-specific symptoms, persistently infected persons have a high risk to develop chronic liver diseases in the course of one or several decades, the most serious outcomes being liver cirrhosis and hepatocellular carcinoma. It is this property of HCV infection and its high prevalence that explain the high medical relevance of this pathogen. Moreover, therapeutic options are limited and there is no prophylactic vaccine in sight [4],[5].

The genome of HCV is a single strand RNA of positive polarity [6]. This RNA has a length of about 9,600 nucleotides and a very simple organization with only one long open reading frame. It is flanked by non-translated regions at the 5′ and 3′ end of the genome that are required for RNA translation and replication. The open reading frame encodes for an about 3,000 amino acids long polyprotein that is cleaved co- and post-translationally by cellular and viral proteases into 10 different products. The structural proteins core, envelope protein 1 (E1) and E2 that build up the virus particle reside in the N-terminal region of the polyprotein and they are processed by host cell signalases and by signal peptide peptidase. C-terminal of E2 is the p7 protein that is required for virus assembly, release [7],[8] and for infectivity in vivo [9]. The remainder of the polyprotein is processed into the nonstructural proteins 2 (NS2), NS3, NS4A, NS4B, NS5A and NS5B. NS2 is a cysteine protease that cleaves at the NS2-3 site and that in addition is required for virus production [7]. NS3 is a bifunctional molecule that carries a serine-type protease activity in the N-terminal domain and a NTPase/helicase activity in the remainder. NS4A is a co-factor of the NS3 protease whereas NS4B is required for the induction of membrane alterations that probably serve as the scaffold for the formation of the membrane-associated replication complex. NS5A is an RNA binding protein assumed to be involved in some step of viral RNA replication and NS5B is the RNA-dependent RNA polymerase.

By using a fully permissive cell culture system that supports the production of infectious HCV particles, we and others have recently gained first insights into the mechanisms underlying HCV particle assembly [10]–[12]. A key player of this process is the core protein. It is composed of an RNA binding domain, a domain for lipid droplet (LD) targeting (domain 2) and a very hydrophobic C-terminal domain (domain 3) that serves as the signal sequence of the C-terminal E1 protein [13],[14]. Building on earlier observations that core protein accumulates on the surface of LDs [15],[16] it was proposed that LDs are involved in the formation of infectious particles. A model was put forward by which core protein recruits the HCV replication complex (RC) to the surface of LDs where virus particle formation may be triggered [10]. Accumulation of core protein on the surface of LDs requires proteolytic removal of domain 3 by signal peptide peptidase [17]. This domain usually anchors the core protein to the ER membrane bilayer and thus precludes mobilization onto the surface of LDs that are surrounded by a monolayer membrane. However, upon removal of domain 3, core becomes mobile and this mobility critically determines efficient virus assembly [11],[12].

Since the core protein accumulates on the surface of LDs whereas the viral RC probably resides on ER or ER-derived membranes, nucleocapsid formation requires the translocation of the viral genome from the RC to core proteins. Cell culture adaptive mutations that enhance virus titres by up to several orders of magnitude without affecting RNA replication have been mapped to the nonstructural proteins constituting the RC. This led to the suggestion that the RC or non-structural proteins may play a role in this putative transfer of the viral genome to the core [18]–[21]. Indeed, one hot-spot of adaptation was found within NS5A arguing that this nonstructural protein may be an element involved in HCV assembly [21].

NS5A is an RNA binding phospho protein composed of 3 domains that are separated by trypsin-sensitive low complexity sequences (LCS I and LCS II) and an N-terminal amphipathic alpha-helix that stably anchors the protein to intracellular membranes [22]–[24]. According to the X-ray crystal structure of domain I, it forms a dimer with a claw-like shape that can accommodate a single strand RNA molecule [25]. The role of domain II and domain III of NS5A in the HCV replication cycle is unknow. However replication enhancing mutations were mapped to a region spanning the C-terminal part of domain I and LCS I arguing that these sequences are important for efficient RNA replication [26],[27]. In contrast, domain III can be deleted or replaced by green fluorescent protein with no dramatic effect on RNA replication [28],[29].

Taking advantage of a highly efficient HCV particle production system, in this study we identified a novel function of NS5A and demonstrate its crucial role in infectious virion assembly. Our data specifically identify domain III in NS5A as a key element in particle formation and provide insight how mutations in this domain lead to accumulation of core protein on LDs and thus may contribute to pathogenesis.

## Results

### Impact of NS5A deletions on HCV RNA replication

**Figure 1:**
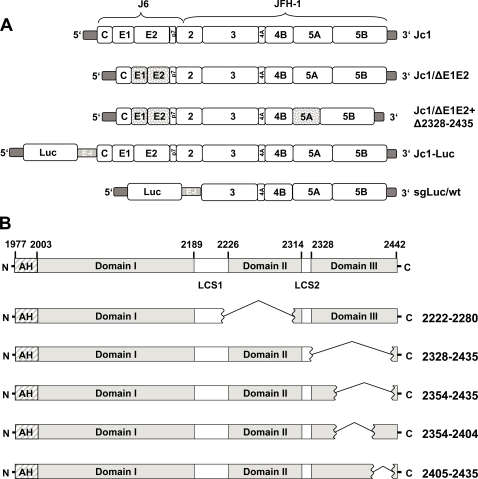
Schematic representation of constructs used in this study. (A) Structure of chimeric HCV genomes consisting of the coding region of core up to the first transmembrane segment of NS2 derived from the genotype 2a isolate J6CF and the remainder of the JFH-1 isolate. Jc1/ΔE1E2 carries an in-frame deletion of 350 codons within the E1-E2 coding region (indicated by dotted boxes). Jc1/ΔE1E2+Δ2328-2435 carries an additional deletion of domain III of the NS5A coding sequence. The structure of the genomic luciferase reporter virus genome is drawn below. It is a bicistronic RNA with the first cistron encoding the firefly luciferase gene (Luc) expressed via the HCV IRES whereas the polyprotein is expressed via the IRES of the encephalomyocarditis virus (E–I). This construct design is analogous to the one of the subgenomic helper RNA (sgLuc/wt; schematic in the bottom). However, it lacks the coding region from core to NS2 and was used for trans-complementation assays. (B) Domain structure of NS5A (upper panel) and deletion mutations in domains II and III (lower panels). For details see text. The structures of the deletion mutants lacking all or parts of domain II or domain III are shown below and their designation is given in the right. Numbers indicate the last and the first amino acid position flanking the deletion or the N- and the C-terminus of JFH-1 NS5A.

**Figure 2:**
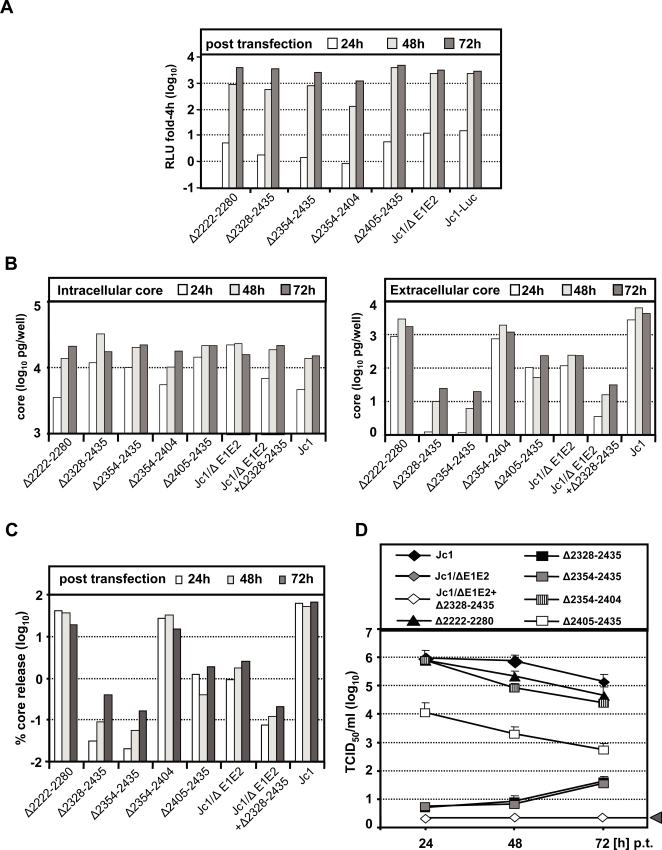
Transient replication of NS5A deletion mutants and release of virus from cells transfected with these genomes. (A) Huh7-Lunet cells were transfected with luciferase reporter virus constructs specified in the bottom. Cells were lysed at given time points after transfection and luciferase activity was determined. A representative experiment of three independent repetitions, each value measured in duplicate is shown. Note that the luciferase reporter genomes have a delayed replication kinetic compared to reporter-free genomes [56] and that Huh7-Lunet cells support HCV spread only poorly [57]. Therefore, luciferase levels in transfected cultures are not affected by virus release and spread and thus reflect almost exclusively RNA replication. (B) Kinetic of intracellular accumulation of core protein (left panel) and release of core protein (right panel) from Huh7-Lunet cells transfected with reporter-free Jc1 constructs. Core amounts were determined by using core-specific ELISA. Intracellular core amounts were normalized to the 4h value that reflects transfection efficiency. A representative experiment of at least three independent repetitions is given in each panel. (C) Efficiency of core protein release from cells transfected with reporter-free Jc1 or given mutants. The percentage of released core protein in relation to total core protein (the sum of intra and extracellular core protein) was calculated for each time point. A representative experiment of three independent repetitions is shown. (D) Release of infectivity from cells transfected with reporter-free Jc1 or mutants specified in the top by using a limiting dilution assay. Cell free culture fluids were harvested at different time points after transfection and titrated to determine viral infectivity of the indicated genomes.The grey bar represents the detection limit of the assay.

To identify the role of domains II and III of NS5A in the viral replication cycle we utilized the chimeric Jc1 genome and inserted a series of in frame deletions into the NS5A coding region (Figure 1). Since NS5A is a component of the HCV replication machinery, we first assessed the impact of these mutations on RNA replication in the context of a well established luciferase reporter virus genome (Jc1-Luc; Figure 1A). In vitro transcripts of these Jc1-Luc constructs were transfected into highly permissive Huh7-Lunet cells in parallel with the Jc1-Luc wild type genome and a deletion mutant that lacked part of the envelope glycoproteins (Jc1-Luc/ΔE1E2). RNA replication was monitored by determination of luciferase activity at 4, 24, 48 and 72 h post transfection. The 4 h value was used for normalization and allowed correction for different transfection efficiencies [27]. As shown in Figure 2A, a deletion spanning almost the complete domain II and retaining only the C-terminal 35 amino acid residues did not affect RNA replication (mutant Δ2222-2280). However, complete removal of domain II abrogated RNA replication (not shown) arguing that the extreme C-terminal sequence of domain II is essential for RNA replication. Complete or partial deletion of domain III of NS5A had no significant impact on RNA replication. Although mutants Δ2328-2435, Δ2354-2435 and Δ2354-2404 showed a clearly reduced replication level at 24 h post transfection, this was overcome 48 h or at latest 72 h after transfection arguing that these deletions had a rather moderate effect on RNA replication (Figure 2A). Analogous results were obtained by using reporter-free Jc1 constructs for transfection and RNA determination by Northern-blot (not shown). Based on these results we concluded that most of domain II (Δ2222-2280) and the complete domain III (Δ2328-2435) are not essential for RNA replication even though some of the deletions in domain III clearly delayed RNA replication kinetics.

### Block of HCV particle assembly by deletion of NS5A domain III

In the light of these surprising results we argued that domain II and domain III may have some other function, most notably in virus assembly and release. Given the impaired capacity of the luciferase reporter genomes to support infectious virus production, in all subsequent experiments only reporter-free constructs were used (Figure 1A). To determine the impact of the NS5A deletion mutants on virus production we quantified core protein amounts in cells transfected with the various mutants and in the corresponding culture supernatants at different time points after transfection by using a core-specific ELISA (Figure 2B, left and right panel, respectively). To account for different efficiencies of core protein accumulation within cells and core protein release, we also determined the relative core release as the ratio of core amounts detected in the culture supernatant to total core protein amounts (intra- and extracellular core) (Figure 2C). Cells transfected with Jc1 released about 50–60% of total core protein into the supernatant. In contrast, relative core protein amounts released from cells that had been transfected with domain II deletion mutant Δ2222-2280 were somewhat reduced and ranged between 40% of total core at early time points after transfection and only 20% 72 h post transfection (Figure 2C). In case of the domain III deletion (Δ2328-2435) extracellular core was almost undetectable arguing for an important role of this NS5A domain for virus release. To map the region within this domain required for infectious particle production three smaller deletions were generated. Retention of the N-terminal 26 amino acid residues of domain III did not restore core release (Δ2354-2435). Inclusion of the 77 N-terminal residues (Δ2405-2435) restored core release to only about 0.5–1% of the wild type (Figure 2C). However, when we retained the N-terminal 26 and the C-terminal 38 amino acid residues, core release comparable to wild type level was observed (Δ2354-2404). These results suggest that the C-terminal region residing between amino acid residues 2404 and 2435 of domain III are crucial for virus production.

In order to study the impact of the deletions in domains II and III of NS5A on assembly and release of infectious HCV particles, infectivity titers in the culture supernatant at 24, 48 and 72 h after transfection with the various mutants, the Jc1 wild type or the envelope glycoprotein deletion mutant (Jc1/ΔE1E2) were determined by using TCID_50_ assays. As shown in Figure 2D, infectivity titers of the wild type were in the range of 10^6^ TCID_50_/ml at early time points after transfection whereas no infectivity was detected at all time points in case of Jc1/ΔE1E2. Infectivity titers obtained with the domain II deletion mutant that still supports core release (Δ2222-2280) were clearly reduced 48 and 72 h p.t. as compared to wild type and this reduction correlated with the reduction of core release (Figure 2C). In agreement with the strongest impairment of core release, almost no infectivity could be detected in supernatants of cells that had been transfected with the two domain III mutants carrying the largest deletions (Δ2328-2435 and Δ2354-2435). Deletion of amino acid residues 2354–2404 or 2405–2435 resulted in about 10-fold or 100-fold lower titers as compared to the Jc1 wild type, respectively (Figure 2D). These results were fully confirmed when we analyzed the analogous luciferase reporter virus genomes (Figure 2A) by determining luciferase transduction efficiency upon infection of naive Huh7.5 cells (not shown).

During these analyses we noted that cells transfected with Jc1/ΔE1E2, which lacks part of the envelope glycoprotein ectodomains, still released well detectable amounts of core protein, but no infectivity. We therefore assumed that this core release is either non-specific, for instance due to cytotoxicity, or that part of the core protein can be released independent from the envelope glycoproteins. To address these possibilities, we constructed a double mutant which contained both the deletion in the E1E2-coding region and the largest deletion in domain III of NS5A (Jc1/ΔE1E2/Δ2328-2435). Interestingly, cells transfected with this mutant released much lower amounts of core protein as compared to Jc1/ΔE1E2 (Figure 2B, C), arguing that a fraction of core protein can be released independent from functional envelope glycoproteins, but still in an NS5A-dependent manner.

### Impact of NS5A domain III deletions on HCV particle release

**Figure 3:**
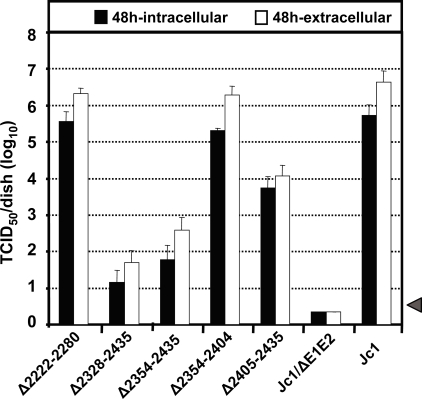
Efficiency of virus assembly and release of Jc1 carrying deletions in NS5A. Huh7-Lunet cells were transfected with the genomes specified in the bottom and 48 h post transfection total infectious particle production was determined. Supernatants and cells were treated by freeze-thaw cycles and viral infectivity was determined by a limiting dilution assay. Mean values of four independent experiments are shown and the standard deviations of the means are presented. The grey bar represents the detection limit of the assay.

The results described above clearly show that domain III plays a primary role in the production of infectious HCV particles, but do not address the question whether assembly or release of these particles is affected. In case of a block of particle release, we would expect an accumulation of cell-associated (intracellular) infectious particles and thus a change of the ratio of extra- over intracellular infectivity. To address this possibility intracellular infectivity titers were compared to the corresponding supernatants. In case of the Jc1 wild type, about 90% of total infectivity was released into the supernatant and only 10% was detectable in transfected cells (Figure 3). A similar ratio was found with the domain II deletion mutant (Δ2222-2280) and the deletion mutation retaining the 26 N-terminal and 38 C-terminal amino acid residues of domain III (Δ2354-2404). In case of the two largest domain III deletion mutants (Δ2328-2435 and Δ2354-2435) intra- and extracellular infectivity titers were reduced by up to 4 orders of magnitude arguing that primarily, if not exclusively, infectious particle assembly was affected. No significant change of extra- over intracellular infectivity was found with the smallest deletion mutant lacking 31 amino acid residues close to the C-terminus of domain III (Δ2405-2435), but titer reduction was much less pronounced as compared to the two largest deletion mutants. These findings were confirmed for cells and supernatants harvested 72 h post transfection (data not shown). We therefore conclude that the deletion mutations residing in domain III affected assembly of infectious virus particles and had little or no effect on virus release.

### Influence of deletions in domain II and III on the phosphorylation status of NS5A

**Figure 4:**
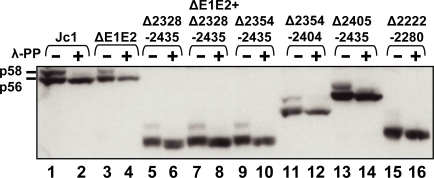
Analysis of the phosphorylation status of NS5A expressed from Jc1, Jc1/ΔE1E2 and Jc1-derived NS5A mutants. Huh7-Lunet cells were transfected with the indicated genomes and lysed in RIPA buffer 72 h post transfection. Cell lysates were treated with aceton/methanol and one-half of the precipitated protein was incubated with λ-phosphatase whereas the other half was mock treated in phosphatase buffer. Both samples were separated by 8% SDS-PAGE and NS5A was detected by Western blot. The positions of p56 and p58 are indicated in the left.

Having confirmed an important role of NS5A for virus assembly, we assumed that NS5A might regulate the formation of HCV particles in a phosphorylation dependent manner and that the deletions we had inserted impact the phosphorylation status of this protein. To test this hypothesis we analyzed the phosphorylation status of the different truncated NS5A proteins by one-dimensional SDS-PAGE after phosphatase treatment. Huh7-Lunet cells were transfected with the different HCV genomes and harvested 72 h after transfection for Western blot analysis (Figure 4). In case of Jc1 the two phosphorylated variants of NS5A, the p56 basal phosphorylated form and the p58 hyperphosphorylated form, were readily detectable. After dephosphorylation of the proteins with λ-phosphatase, the p58 variant disappeared and the p56 variant migrated slightly faster than the non-treated protein, showing that both forms were phosphatase sensitive. For all domain III mutants, a basally and a hyperphosphorylated variant of NS5A were detected. The only mutant that showed a clear reduction in the amount of hyperphosphorylated NS5A carried the deletion in domain II (Δ2222-2280). Since this deletion did not significantly affect RNA replication and particle production, our results suggest that either very low amounts of hyperphosphorylated NS5A (not detectable in this assay) are sufficient for RNA replication and particle assembly or that hyperphosphorylated NS5A is dispensable for these processes.

### Deletion of domain III of NS5A results in a loss of colocalization of NS5A and core protein on lipid droplets

**Figure 5:**
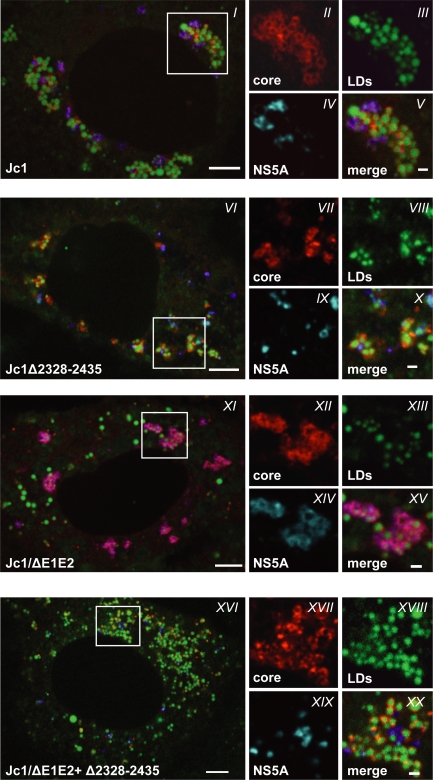
Deletion of domain III of NS5A abolishes NS5A colocalization with core on LDs. Huh7-Lunet cells were transfected with Jc1 (panel *I* to *V*), Jc1/Δ2328-2435 (panel *VI* to *X*), Jc1/ΔE1E2 (panel *XI* to *XV*), and Jc1/ΔE1E2+Δ2328-2435 (panel *XVI* to *XX*) and fixed 72 h post transfection. Fixed cells were analyzed for the subcellular localization patterns of core (red) and NS5A (blue) by immunofluorescence whereas LDs were stained with BODIPY493/503 (green). Images were generated with a spinning disk confocal and they represent single optical planes. The locations of the 4 cropped sections shown for each of the constructs in the right are boxed in each overview panel. Scale bars represent 5 µm and 1 µm in single cell and cropped sections, respectivetly.

We have recently shown that the core protein appears to recruit the HCV replicase and the RNA genome to LDs, which may be the site for the assembly of infectious HCV particles [10]. Since deletions in domain III very much impair particle assembly, we assumed that these mutations may affect either the localization of NS5A on LDs *per se* or the core-dependent recruitment of NS5A to LDs. To address these possibilities we first studied the subcellular localization of the HCV proteins in cells transfected with the NS5A mutants or the Jc1 wild type or the E1-E2 deletion mutant. Seventy two hours after transfection cells were fixed and stained for NS5A, core protein and LDs. In case of Jc1 wild type, core protein accumulated on LDs (Figure 5, panel *II*). In addition, low amounts of NS5A were also found on LDs and this NS5A species colocalized with the core protein (panel *IV* and *V*, respectively). In agreement with our earlier report [11], core accumulation on LDs was very much pronounced when assembly was blocked at a rather late stage, which we achieved here by partial deletion of the ectodomains of E1 and E2 (Jc1/ΔE1E2) (Figure 5, panel *XII*). Concommitant with core protein accumulation, we also found an accumulation of NS5A on LDs and a perfect colocalization with the core protein (panel *XIV* and *XV*, respectively). Interestingly, in case of the mutant with the strongest impact on infectious particle assembly (Jc1/Δ2328-2435), both NS5A and core protein still accumulated on the surface of LDs (Figure 5, panel *VII*, *IX*), but their colocalization on the same LDs was no longer detectable (panel *X*). The same phenotype was found with the double deletion mutant Jc1/ΔE1E2+Δ2328-2435 (Figure 5, panel *XVI* to *XX*).

**Figure 6:**
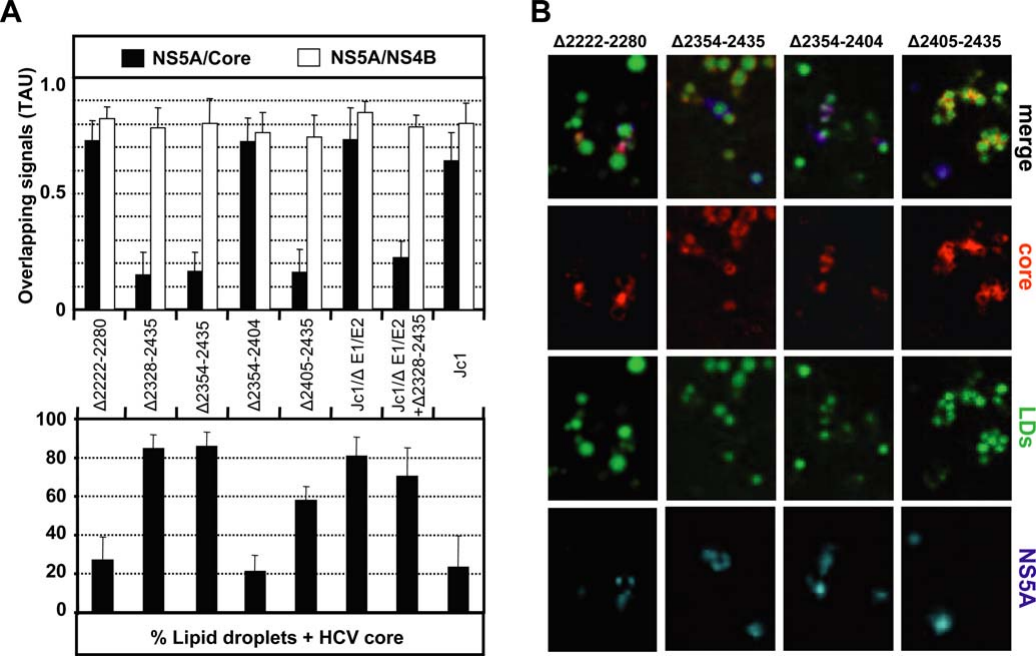
Mutations in domain III of NS5A impairing virus assembly also impair colocalization of NS5A with core protein on LDs. (A) The quantitation of colocalization between NS5A and core protein, or NS5A and NS4B in a population of 50 cells per construct is shown in the upper panel. For the measurement of the degree of colocalization, the correlation coefficients between the signal obtained for the respective proteins were calculated by using the plugin ‘Intensity correlation analysis’ of Image J. The lower panel summarizes the association of core protein and LDs in cells transfected with the constructs specified between both panels. For calculation of the percentage of core protein associated with LDs about 2,000 to 2,500 LDs were counted for each construct and colocalization with core protein was analyzed by using the plugin ‘RG2B colocalization’ of Image J. Error bars in both panels represent the standard errors of the means of 10 to 15 cells per construct. (B) Representative confocal immunofluorescence images of cells transfected with the constructs as specified in panel A.

A more quantitative analysis of core and NS5A colocalization on LDs fully confirmed this observation (Figure 6A, upper panel). Including the other NS5A mutants in the analysis, a clear correlation between core – NS5A colocalization on LDs and the efficiency of core release was detected. Most notably those deletions in domain III of NS5A that severely reduced the assembly of infectious particles also reduced core – NS5A colocalization on LDs (Figure 6A, upper panel and Figure 6B). Interestingly, when we determined the colocalization of all these NS5A proteins with NS4B, which is most likely an integral component of the HCV replication complex, none of the mutations affected the colocalization with NS4B (Figure 6A, upper panel). This observation is consistent with the result that the NS5A mutants support HCV RNA replication, in most cases comparable to wild type levels. The data thus imply that NS5A may exist in two different complexes. One complex containing the core protein and presumably responsible for particle assembly, and one complex containing NS4B and probably representing the (membranous) replication complex. Alternatively, NS5A may exist as a component of the replicase complex only, but this complex is no longer recruited to the core protein on the surface of LDs in case of the mutant lacking domain III.

Another interesting observation emerged when we quantified the core protein accumulating on the surface of LDs in cells transfected with the various HCV genomes. As summarized in the lower panel of Figure 6A, core protein accumulation was highest for those NS5A mutants that do not support production of infectious virus particles (Δ2328-2435 and Δ2354-2435). In contrast, overall only low amounts of core co-localized on the surface of LDs in case of the Jc1 wild type, even though in a few cells colocalization was extensive (one example is shown in Figure 5). Likewise, in cells transfected with the NS5A mutants Δ2222-2280 and Δ2354-2404 that both support efficient virus production (see also Figure 2D), only about 20% of LDs colocalized with core. An intermediate phenotype was found in case of the Δ2405-2435 NS5A mutant that supported about 100-fold lower virus titers as compared to wild type. This result correlates with the model that core protein residing on the surface of LDs is involved in virion assembly and that inefficient assembly results in an accumulation of core on LDs. In agreement with that assumption, core protein accumulation on the surface of LDs was also observed with the envelope deletion mutant (Jc1/ΔE1E2). However, in this case we observed a colocalization of core and NS5A, unlike the assembly-incompetent NS5A mutants.

**Figure 7:**
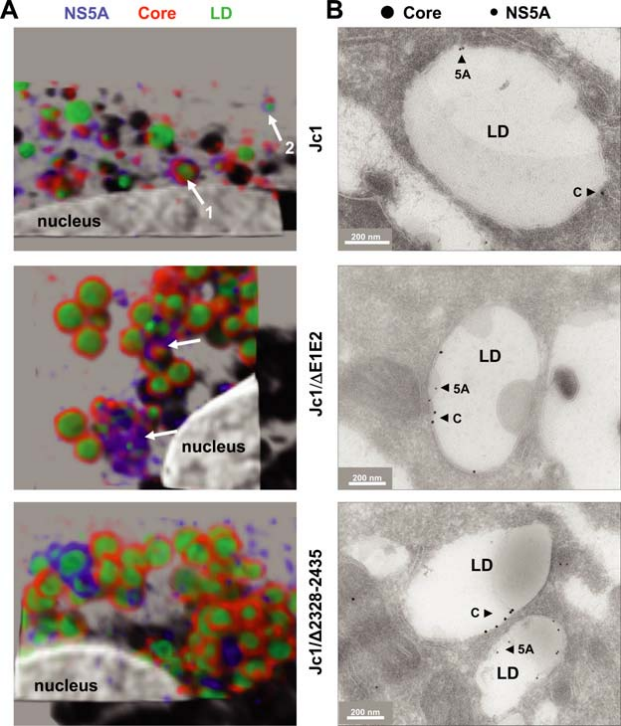
Disruption of NS5A – core colocalization on LDs by deletion of NS5A domain III. (A) Cells transfected with the Jc1 wild type, the envelope deletion mutant (Jc1/ΔE1E2) or the domain III deletion mutant (Jc1/Δ2328-2435) were grown on coverslips and double labeled with core- and NS5A-specific antibodies; LDs were stained with BODIPY493/503 (green) and the nuclei with DAPI. Subcellular distribution of HCV proteins on LDs was analyzed by confocal fluorescence microscopy. For enhanced clarity of 3-dimensional distribution of markers, the image stacks were deconvolved as described in Materials and Methods. The deconvolved z-stacks are shown in a projection including a shadow cast to create a 3-dimensional impression. Arrows refer to LDs with core – NS5A colocalization. Numbers and arrows in the upper and middle panel indicate colocalization patterns as described in the text. (B) Detection of HCV core and NS5A in transfected cells by using immuno electron microscopy. Cells transfected with the Jc1 wild type, the envelope deletion mutant and the NS5A mutant were grown in 10 cm^2^ cell culture dishes and fixed after 48 hours. Thawed cryosections were prepared (as described in Materials and Methods) and double-labeled with antibodies to HCV NS5A (10-nm-diameter protein A-gold) and HCV Core (15-nm-diameter protein A-gold). *Bars*, 200 nm. LD, lipid droplet. A quantification of the immunolabeling is summarized in Table 1.

Although the data described thus far suggested that NS5A mutants lacking domain III no longer colocalized with core on the surface of LDs, the level of resolution achieved with these assays did not allow us to draw a firm conclusion about the localization of NS5A and core protein relative to each other. We therefore analyzed cells transfected with the NS5A domain III deletion mutant Jc1/Δ2328-2435 in parallel to cells transfected with Jc1 wild type or the envelope deletion mutant (Jc1/ΔE1E2) by using deconvolution of images generated by spinning disk confocal microscopy. As shown in Figure 7A, in Jc1 transfected cells the core protein localized directly to the surface of the droplets, often in association with NS5A (structure 1 in Figure 7A, upper panel). In some other cases NS5A was directly associated with the surface of LDs whereas the core protein accumulated at a distinct spot on the droplet surface which may correspond to the loading site of core onto LDs [12] (structure 2 in Figure 7A, upper panel). In case of the envelope deletion mutant, core and NS5A still colocalized (arrows in Figure 7A) and core staining was much more intense reflecting an accumulation of core protein on the surface of LDs due to an assembly defect. A comparably high core accumulation on LD surfaces was found with the NS5A domain III deletion mutant consistent with the assembly block (Figure 7A, lower panel). Most importantly, however, core and NS5A staining no longer co-localized arguing that domain III in NS5A is required for colocalization with core.

These results were fully confirmed by immuno electronmicroscopy (Figure 7B and the quantification summarized in Table 1). Core and NS5A colocalized on the surface of the same LD in cells transfected with the Jc1 wild type (upper panel). In contrast, in case of the domain III deletion mutant, only very rarely core and NS5A were found on the surface of the same LD even though the antigen amounts were much higher as compared to the wild type (Figure 7B, lower panel and Table 1). However, core – NS5A colocalization was detected in case of the envelope gene deletion mutant. In summary these data clearly show that domain III of NS5A is required for colocalization with core on the surface of LDs. In the light of an earlier report [10] the results thus imply that domain III-dependent recruitment of NS5A (or the viral replicase) to core-containing LDs is required for HCV particle assembly.

**Table 1:** Average amount of gold particles on the membrane of LDs in cells that had been transfected with Jc1 wild type or Jc1/ΔE1E2 or Jc1/Δ2328-2435 (exemplified by Figure 7B).

	Jc1	Jc1/ΔE1/E2	Jc1/Δ2328-2435
Anti-core	1.21	3.48	3.16
Anti-NS5A	2.06	2.59	2.46

The data are based on two different labeling experiments, two grids per experiment considering 20 lipid droplets per grid. Only the labeling on the membrane around the LD was considered, and not labeling on adjacent vesicles or the ER membrane. Statistically significant differences were only observed in case of core labeling between Jc1 wild type and the two mutants (p-value <0.0001 as determined by t-test), but not between the two mutants. Likewise, labeling density of NS5A did not differ between the three constructs in a statistically significant manner.

### Rescue of HCV particle assembly by trans-complementation of NS5A deletion mutants

**Figure 8:**
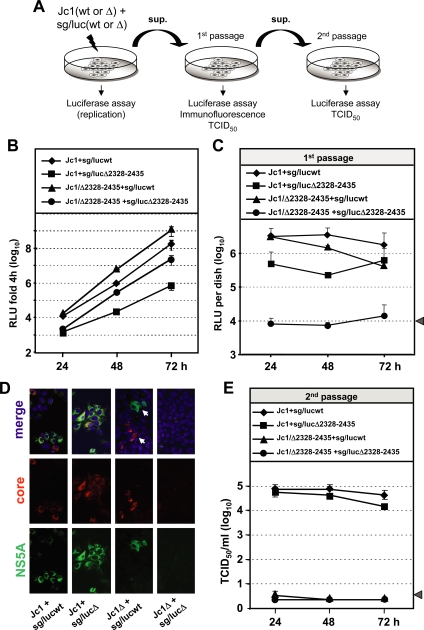
Particle assembly of NS5A mutants can be rescued by trans-complementation. (A) Schematic of the experimental approach. Genomic (Jc1) wild type or mutant RNA was cotransfected with one of the given helper RNAs (sg/lucwt or sg/lucΔ2328-2435). Replication of the helper was determined by luciferase assay. Release of infectious particles from transfected cells was determined by infection of naïve Huh7.5 cells with supernatants (sup.) from transfected cells and subsequent analysis of infected cells (1^st^ passage) by luciferase assay, immunofluorescence analysis or TCID50 assay. Infectivity release from these infected cells was determined by subsequent inoculation of naïve Huh7.5 cells (2^nd^ passage) with the corresponding supernatants (sup.) and infection was determined by luciferase assay or TCID_50_ assay. (B) Transient replication of sg/luc helper RNAs 24, 48 and 72 h after co-transfection with Jc1 wild type or the Jc1/Δ2328-2435 mutant. (C) Supernatants from transfected cells (panel B) were harvested 24, 48 and 72 h post transfection and used for inoculation of naïve Huh7.5 cells. The level of trans-packaged or trans-complemented helper RNA was determined by luciferase assay with cells 72 h after inoculation. (D) Supernatants from transfected cells (panel B) were harvested 48 h post transfection and used for inoculation of naïve Huh7.5 cells. Seventy two hours after inoculation cells were fixed and NS5A or core protein were detected by immunofluorescence. Nuclei of cells were stained with DAPI. (E) Supernatants from cells after first passage were harvested 72 h post infection and used to inoculate naïve Huh7.5 cells. Infectivity in the supernatant of these cells was determined by TCID_50_ assay with supernatant harvested 72 h post inoculation. Values shown in panels B, C and E represent the mean of two independent experiments, each measured in duplicate and the error bars.

NS5A is a component of the viral replicase and therefore involved in RNA replication. Recently we showed that replication-incompetent NS5A mutants could be rescued by trans-complementation [30]. Having now found that NS5A is involved in HCV assembly, we were interested whether this NS5A function can also be restored by trans-complementation. To this end we established a trans-complementation assay based on the co-transfection of a Jc1 genome carrying the deletion in domain III of NS5A (Δ2328-2435) with a subgenomic luciferase-helper RNA that lacks the region encoding core to the C-terminus of NS2 (Figure 1A). Replication of this helper RNA was determined by luciferase assay (Figure 8B). In addition, for control purposes we included cotransfections of Jc1 wild type with the helper RNA as well as cotransfections of either of the Jc1 constructs with a defective helper RNA that carried the large domain III deletion in NS5A (Figure 8A). Depending on the particular combination of the constructs, different outcomes were possible. Upon transfection of cells with the wild type helper RNA and the Jc1 wild type genome, we would expect the generation of infectious Jc1 particles containing the full length wild type genome or, in case of trans-packaging, the subgenomic helper RNA. In fact, upon inoculation of naive Huh7.5 cells with supernatants from such transfected cells, high level luciferase expression was detected indicating the formation of virus-like particles which had encapsidated the subgenomic helper RNA as a result of trans-packaging (Figure 8C). Moreover, inoculated cells expressed high amounts of wild type NS5A and core protein indicating efficient infection with Jc1 wild type particles (Figure 8D). Upon cotransfection of cells with Jc1 wild type and the defective helper RNA (sg/lucΔ2328-2435), infectious virus-like particles were generated that also transduced the luciferase gene (Figure 8C). Formation of these particles was most likely due to trans-complementation of the defective NS5A of the helper RNA by wild type NS5A expressed from Jc1 and subsequent trans-packaging of the helper RNA into virus-like particles. In addition, infectious Jc1 particles were produced as determined by high level core and NS5A expression in cells inoculated with supernatant of the cotransfected cells (Figure 8D). The epitope recognized by the NS5A-specific antibody used for this immunofluorescence staining resides in domain III thus allowing selective detection of wild type NS5A protein but not the domain III deletion mutant.

In case of cells cotransfected with the Jc1-NS5A mutant (Jc1/Δ2328-2435) and the wild type helper RNA (sg/lucwt) efficient transduction of the luciferase gene was observed arguing for trans-packaging of the helper RNA by the structural proteins provided by the Jc1 mutant (Figure 8C). In addition, core expression in infected cells was observed arguing for a rescue of the Jc1 mutant by trans-complementation that was mediated by the intact NS5A protein provided by the helper (Figure 8D). This assumption is supported by the fact that most cells expressing well detectable amounts of core protein did not express wild type NS5A protein indicating that these cells indeed had been infected with the Jc1-NS5A mutant. In contrast, cells expressing wild type NS5A (expressed from the transduced helper RNA) did not express core protein supporting the notion that these cells had been infected with virus-like particles that had packaged the subgenomic helper RNA.

Finally, in supernatants of cells cotransfected with the Jc1 mutant (Jc1/Δ2328-2435) and the defective helper RNA (sg/lucΔ2328-2435) no infectivity was detected, neither by luciferase assay (Figure 8C) nor by immunofluorescence (Figure 8D) and TCID_50_ assay (not shown). This result supports the notion that the NS5A protein expressed from the helper RNA carrying the deletion in domain III indeed is unable to support virus assembly.

To exclude the possibility of RNA recombination between the Jc1-NS5A mutant and the wild type helper RNA resulting in a wild type Jc1 genome, supernatants from primary infected cells were passaged (Figure 8A; 2^nd^ passage). Supernatants from such inoculated cells were then analyzed for infectivity by using luciferase assay (not shown) and TCID_50_ assay (Figure 8E). However, in no case infectivity was detected excluding the possibility that a Jc1 wild type with or without a luciferase gene was generated by RNA recombination.

In conclusion these results demonstrate that also alterations affecting the assembly/virus release function of NS5A can be restored by trans-complementation. The data also show that subgenomic HCV RNAs can be packaged efficiently into infectious HCV-like particles.

## Discussion

A hallmark of the HCV replication cycle is its extraordinary dependance on host cell lipids. Several groups have shown that viral RNA replication is tightly linked to lipid synthesis pathways and sensitive to pharmacological intervention with statins and certain fatty acids [31]–[33]. More recently it became apparent that HCV particle morphogenesis and egress also depend on host cell lipids [34]. Taking advantage of the newly established cell culture system to produce infectious virus particles, evidence was obtained that HCV assembly occurs in close association with LDs [10],[12]. It was suggested that core protein accumulates on the surface of LDs and recruits the RC, and thus the viral RNA, in order to trigger particle formation [10]. However, the mechanisms of RC recruitment and initiation of assembly are not known. In this study, we show that NS5A, in addition to core, is a key element of particle formation. The primary determinant within NS5A is domain III. Interestingly, deletions affecting this domain do not abrogate LD association of NS5A suggesting that LD association is mediated by some other domain(s) within NS5A, the most likely being domain I, eventually in conjunction with the N-terminal amphipathic alpha-helix (10). However, domain III deletions affect colocalization with core protein on the surface of the same LD. This result implies that different types of LDs are formed in case of the mutant: those that contain primarily core protein and those that contain almost exclusively NS5A (Figure 7B). The underlying mechanism is not known but it is possible that core protein has a strong binding affinity to LDs and thus heavily occupies the surface of LDs. This would leave only limited access for NS5A to the surface of such a LD. In case of wild type NS5A facilitated by an (direct or indirect) interaction between NS5A and core, both proteins can accumulate on the surface of the same LD. In case of the domain III deletion mutant, the core – NS5A interaction would be disrupted and thus, this mutant NS5A protein would primarily accumulate on LDs not occupied by core protein. Further studies are necessary to address this possibility.

The fact that deletions in domain III perturb colocalization with core on LDs and at the same time impair infectious virus production provides compelling evidence that both events are linked. The data support a model in which core recruits either NS5A or the NS5A-containing RC via direct interaction with domain III to the assembly site. In agreement with this assumption a direct core – NS5A interaction has been demonstrated in an overexpression system by using coprecipitation and colocalization studies [35],[36]. We also attempted to demonstrate a direct core – NS5A interaction by using infected or transfected cells as well as over-expression systems. Neither by co-immunoprecipitation with or without cross-linking nor by using fluorescence resonance energy transfer assays we were able to demonstrate such an interaction. Differences in the experimental set up as well as differences in the used HCV isolates (genotype 2a (JFH-1) versus genotype 1a and 1b in the previous studies) could account for this discrepancy. However, it is well possible that in a more authentic (virus production) system such a core – NS5A interaction is very unstable and transient. It is also conceivable that within an assembly competent replication system only a minor fraction of total NS5A and core protein is enganged in an interaction. Moreover, once RNA transfer from the RC (or NS5A) to core has been initiated, a self-assembly process driven by core – RNA interaction may take place that no longer depends on an interaction between core and NS5A. It is even possible that core and NS5A do not directly interact but that exposed RNA regions not complexed by NS5A are recognized by core protein residing in close proximity to NS5A and sufficient to trigger assembly. In this respect colocalization of core and NS5A on the surface of the same LD or in close proximity to each other may be sufficient to allow particle formation. Finally, NS5A – core interaction may be indirect and facilitated by host cell factors. Several NS5A binding proteins have been described such as VAP-A/B and VPS35 that are involved in intracellular trafficking or apolipoproteins involved in the formation of lipoproteins [35],[37]. The contribution of these host cell factors for virion formation remains, however, to be determined. It is also unclear why HCV utilizes this unique pathway of virion formation and where exactly envelopement of the nucleocapsid occurs. In agreement with earlier observations [10], we found that E2 also accumulated in close proximity of LDs (N.A., M.Z. T.S. and R.B., unpublished). It is therefore plausible that LDs tightly surrounded by ER membranes are the sites where nucleocapsids form, which could then acquire their envelope via budding into the ER lumen at sites in close proximity of LDs. Further studies are required to address this important question.

At least two important consequences arise from an assembly model that is based on a RC (NS5A) – core interaction. First, specificity of RNA encapsidation would primarily be brought about by protein – protein rather than protein – RNA interaction. While the genome of several viruses, such as e.g. the hepatitis B virus, contain a distinct RNA element that is recognized by a viral protein to mediate selective genome packaging [38], such a packaging signal would not be required for HCV. Second, mutations in NS5A affecting RNA binding or core interaction would interfere with assembly and thus lead to an accumulation of core on the surface of LDs.

It is interesting to note that NS5A, especially the so-called V3 region of domain III is amongst the most variable sequences across the different genotypes and subtypes [39]. Therefore, it is well possible that NS5A variants differ in their efficiency for infectious particle assembly. Inefficient assembly results in an accumulation of core protein on the surface of LDs [11] and intracellular accumulation of core can lead to perturbation of host cell lipid metabolism such as interference with microsomal triglyceride transfer protein activity and very-low density lipoprotein (VLDL) secretion [40]. Thus, both core and NS5A may contribute to pathogenesis, especially HCV-induced steatosis.

In agreement with earlier studies, we found that deletions affecting domain III have no or minimal impact on RNA replication [29],[41]. However, insertion of a heterologous sequence into the coding region of domain III impairs virus production [42]. In the light of the present data the most likely explanation for this phenotype is that the insertion interferes with the assembly function of domain III. To our great surprise we also found that a nearly complete deletion of domain II (Δ2222-2280) has no impact on RNA replication and almost no effect on virion production arguing that domain II serves some other purpose not directly contributing to the replication cycle such as interaction with the host cell. This result is to some extent at variance to a recent report by Tellinghuisen and colleagues [41] showing that a 10 amino acid deletion in domain II (deletion B) overlapping the larger deletion that we describe here (Δ2222-2280) blocks RNA replication. This discrepancy may be due to the different experimental systems used in their and our study [genotype 1b (Con1) derived subgenomic replicons versus JFH-1 derived genomes, respectively]. Moreover, it is interesting to note that only for deletion B Tellinghuisen and colleagues could not identify a single amino acid responsible for the non-replicating phenotype arguing that the complete region rather than a specific amino acid is required for RNA replication. Since the overall amino acid sequence homology of NS5A between Con1 and JFH-1 is about 61%, but only about 50% in case of domain II, it is possible that in JFH-1 other regions of the NS5A sequence are required for efficient RNA replication. Nevertheless, when we deleted the complete domain II, replication of JFH-1 was also completely blocked (not shown). This observation fits to the results by Tellinghuisen and colleagues, who showed that 10 amino acid deletions affecting the C-terminal half of domain II prevent RNA replication completely [41].

By using a cotransfection approach of a NS5A mutant genome with a subgenomic helper RNA we demonstrate that both trans-complementation and trans-packaging occur. Trans-complementation means that the mutant RNA genome that due to a deletion of domain III is not assembly competent can be rescued by providing NS5A (expressed in the context of a replicase) in trans. The result is a virus-like particle that contains the mutant genome and thus upon infection of naïve cells can not spread in culture due to the assembly defect. The molecular mechanisms underlying this trans-complementation are not known and therefore we can only speculate. In one possible scenario mixed replication complexes form composed of the genomic NS5A mutant and the subgenomic helper, allowing wild type NS5A to interact with mutant RNA and thus ‘tagging’ the mutant RNA genome for encapsidation. Whatever the underlying mechanism is, preliminary results suggest that NS5A expressed on its own does not support trans-complementation, but only when NS5A is expressed in the context of the replicase (NS3 to NS5B; N.A., S.K. and R.B. unpublished). Thus, the pre-conditions required to rescue assembly-defective mutants by trans-complementation with NS5A appear to be similar to those required to rescue NS5A mutants that are defective in RNA replication (30).

Apart from trans-complementation we also observed trans-packaging which refers to the encapsidation of the subgenomic helper RNA into virus-like particles. These particles are infectious but due to the subgenomic nature of the encapsidated RNA, no infectious virus progeny is generated and thus there is no spread in cell culture. Trans-packaging has also been described for numerous other viruses including alphaviruses as well as several members of the pesti- and flaviviruses [43],[44]. In these cases trans-packaging has been achieved by several different strategies including packaging defective helper constructs or helper cell lines that stably express the structural genes. Moreover, trans-packaging of subgenomic RNAs has been observed in vivo and is refered to as defective-interfering particles which means the formation of virus-like particles that contain an RNA subgenome and that upon co-infection of a cell with a wild type genome interfere with wild type RNA replication [45]. A recent report suggests that such particles are also formed in HCV-infected patients [46].

In summary, we demonstrate that NS5A is a major determinant for infectious virus production and show that domain III is most critical for this step in the viral replication cycle. In the light of this observation and given the essential role of NS5A for RNA replication, this protein is a novel and promising target for antiviral therapy. Several inhibitors targeting the replication function of NS5A have been described and it will be interesting to determine whether they also impact virus particle production and thus follow a two-pronged mode-of-action.

## Materials and Methods

### Plasmids

Plasmids pFK-Jc1 and pFK-Luc-JFH1 have been described previously (Wakita 2005; Pietschmann 2006). PFK-Jc1/ΔE1E2 that carries a deletion of 350 codons in the E1-E2 coding region (removing amino acids 218–567 of the J6 polyprotein) and the NS5A deletions were generated by PCR-based mutagenesis. All PCR-amplified DNA fragments were analyzed by automated nucleotide sequencing using an ABI 310 sequencer (Applied Biosystems). Big Dye version 1.1 (Applied Biosystems) was used for cycle sequencing according to the manufacturer's protocol. Detailed information about DNA cloning is available in Protocol S1.

### Cell culture and infectivity assays

Huh-7 cell clones Huh7-Lunet [47] and Huh7.5 [48] that both are highly permissive for HCV RNA replication were used for electroporation and infection assays, respectively. Luciferase reporter virus-associated infectivity was determined as described elsewhere [49]. Infectivity of HCV variants lacking a reporter gene was determined by using a limiting dilution assay on Huh-7.5 cells [50] with a few minor modifications. Infected cells were detected by using a JFH1 NS3-specific rabbit polyclonal antiserum as primary antibody (dilution 1∶500) and a peroxidase-conjugated goat anti-rabbit polyclonal antibody (Sigma) as a secundary antibody (dilution 1∶1,000). The tissue culture 50% infectivity dose (TCID_50_) was calculated as described recently [51]. Intracellular infectivity assays as determined with freeze – thaw lysates of transfected cells were performed according to a published protocol [52]. In brief, 48 h post-transfection Huh7-Lunet cells were extensively washed with PBS, scraped off the plate and centrifuged for 5 min at 700×g. Cell pellets were resuspended in 1 ml of DMEM containing 5% FCS and subjected to three cycles of freezing and thawing using liquid nitrogen and a thermo block set to 37°C. Samples were then centrifuged at 10,000×g for 10 min at 4°C to remove cell debris, and cell-associated infectivity was determined by TCID_50_ assay. Culture supernatants from transfected cells were treated in the same way and infectivity was determined in parallel.

### Transient HCV replication and trans-complementation assay

Transient HCV RNA replication assays were performed as described previously [53]. In brief, plasmid DNA was restricted with *Mlu*I and used for in vitro transcription. Ten µg of run-off transcripts were used for electroporation of 4×10^6^ Huh7-Lunet cells that were resuspended in 20 ml culture medium (12 ml in case of freeze and thaw experiments). Two ml aliquots were seeded per well of a 6-well plate and replication was determined by measuring luciferase activity in case of genomes containing the luciferase reporter gene at 4, 24, 48 and 72 h post-electroporation. Since luciferase activity measurable 4 h post transfection is derived from transfected input RNA, these values were used to normalize for transfection efficiency. In case of authentic virus genomes replication was monitored 0, 24, 48 and 72 h post electroporation by Northern blot analysis as described in Protocol S1. For trans-complementation assays, Huh7-Lunet cells were co-transfected with 5 µg of Jc1 genomes and 0.5 µg of helper RNAs containing a luciferase reporter gene (corresponding to a 1∶0.1 molar ratio, respectively). Electroporated cells were seeded as described above and replication of the non-reporter genome was determined by Northern-blot analysis. Transient replication of helper RNAs was determined by luciferase assay 4, 24, 48 and 72 h after electroporation. Values obtained 4 h post electroporation were used to determine the transfection effciency. Supernatants were harvested 24, 48 and 72 h after eletroporation and concentrated three times by using Amicon columns (Millipore, Schwalbach) according to the instructions of the manufacturer. Release of infectious particles from co-transfected cells was determined by TCID_50_ assay by using the concentrated culture supernatants. Replication of trans-packaged subgenomic helper RNA was determined by luciferase assay performed with lysates of Huh 7.5 cells that had been inoculated with the concentrated culture supernatants of co-transfected cells.

### Quantification of HCV core protein by Elisa

Core protein amounts were determined by using the Trak-C Core ELISA (Ortho Clinical Diagnostics) as recently described [51]. Since core protein amounts measurable 4 h after transfection are derived from transfected input RNA, these values were used to normalize for transfection efficiency.

### Protein precipitation, phosphatase treatment and Western blot analysis

Huh7-Lunet cells electroporated with Jc1 genomes were seeded into a 10 cm diameter culture dish. Seventy two hours post electroporation cells were washed two times with ice-cold phosphate-buffer saline (PBS) and harvested by scraping into 500 µl RIPA buffer (50 mM Tris-HCl [pH 7.4], 1% Nonidet P-40, 0.25% sodium desoxycholate, 150 mM NaCl, supplemented with protease inhibitors (1 mM PMSF; 0.001 U/ml aprotinin and 4 µg/ml leupeptin). The cell lysate was divided into two aliquots, mixed with 4 volumes of acidified acetone/methanol and incubated at −20°C over night. Proteins were pelleted by centrifugation at 15,000×g for 15 min, pellets were air dried and resuspended in 200 µl of lambda phosphatase buffer (50 mM Tris HCl [pH 7.5], 100 mM NaCl, 0.1 mM EGTA, 2 mM DTT, 0.01% Brij35) supplemented with 2 mM MnCl_2_ and 0.4% NP-40. One hundred µl of the sample was incubated with 4,000 units of lambda protein phosphatase (Biolabs) at room temperature (RT) for 45 min. Samples were then mixed with SDS-PAGE loading buffer [200 mM Tris-HCl [pH8.8], 5 mM EDTA, 0.1% bromophenol blue (w/v), 10% sucrose (w/v), 3% SDS (w/v) and 2% beta-mercaptoethanol (v/v)], separated by electrophoresis into an 8% polyacrylamide gel and transferred to a PVDF membrane. NS5A was detected by immunobloting using a JFH-1 NS5A-specific rabbit polyclonal antiserum (dilution 1∶1,000) and peroxidase-conjugated goat anti-rabbit polyclonal antibody (dilution 1∶25,000). Bound secondary antibody was detected by using the ECL Plus Western Blotting Detection system (Amersham) according to the instructions of the manufacturer.

### Immunofluorescence analyses and image deconvolution

Transfected Huh7-Lunet cells were seeded into 24 well-plates containing glass coverslips. Seventy two hours after electroporation, cells were washed twice with PBS, fixed with 4% paraformaldehyde in 150 mM sodium cacodylate buffer [pH 7.5] for 15 min at RT and permeabalized with digitonine (50 µg/ml) for 5 min at RT. Permeabilzed cells were washed twice with PBS and blocked with PBS containing 5% (w/v) bovine serum albumine (Sigma) for 30 min at RT. NS5A was detected by using a NS5A-specific monoclonal antibody (Austral Biologicals, San Ramon, CA) at a dilution of 1∶200; core and NS4B were detected with monospecific rabbit polyclonal antisera C-830 (dilution 1∶200) or serum #86 (dilution 1∶100), respectively. After 1 h at RT, cells were washed three times with PBS and incubated with a 1∶1,000 dilution of Alexa 488, 546 or 647-conjugated secondary antibody (Invitrogen, Molecular Probes) in PBS - 5% BSA for 1 h in the dark. LDs were stained with 20 µg/ml of BODIPY493/503 (Invitrogen, Molecular Probes) during secondary antibody incubation. Cells were washed once with PBS, incubated for 1 min with a 1∶5,000 diluted solution of 4′, 6′-diamidino-2-phenylindole dihydrochloride (DAPI)-PBS, and immediately washed four times for 10 min with PBS. Cells were mounted on glass slides with Slow-Fade Gold Antifade Reagent (Invitrogen, Molecular Probes). For double staining images were acquired on a Nikon C1Si spectral imaging confocal laser scanning system on a TE-2000 E equipped with 60× Objective (NA 1.4). For 3-D reconstruction of samples stained with 3 or 4 markers, cells were imaged on an Ultraview ERS spinning disk (PerkinElmer Life Sciences) on a Nikon TE2000-E inverted confocal microscope equipped with a Plan-Apochromat VC 100X lens (NA 1.4). Channels were recorded sequentially onto an EM-CCD camera by using an emission discrimination option in the following order: 647/700, 568/610, 488/510, 405/440 (emission/excitation). For deconvolution, optical slices were acquired at 0.15-µm Z spacing resulting in a stack of 30–40 optical slices per cell. Colocalization of fluorescence signals was evaluated quantitatively for Pearson's correlation coefficient (R_r_) by using the plugin ‘Intensity Correlation Analysis’ of the ‘Image J’ software. For each sample 50 cells were analyzed. Core-LD colocalization was calculated by counting the total number of LDs in a cell and the number of those LDs with an overlapping core protein signal. The plugin ‘RG2B’ colocalization software of Image J was used to detect overlapping signals. Deconvolution of image z-stacks was performed based on a theoretical point spread function by using Huygens Essential software (v. 3.0, Scientific Volume Imaging BV). The 3D projections of deconvolved images were reconstructed with the help of the simulated fluorescence process volume rendering algorithm of the Huygens Essential software. In case of trans-complementation assays (Figure 8D), infected Huh 7.5 cells were treated as described above, but NS5A was detected with monoclonal antibody 9E10 [50] and images were acquired with an inverted fluorescence microscope (Leica, Germany).

### Immunoelectron microscopy

For immuno-EM Huh7.5 cells grown in 10 cm^2^ dishes were transfected with viral RNA as described above. Forty eight hours post transfection the cells were fixed in 4% paraformaldehyde, 0.1% glutaraldehyde, and 1% acrolein in PHEM buffer (240 mM Pipes, 100 mM Hepes, 8 mM MgCl2, 40 mM EGTA; pH 6.9). Cells were scraped off the dish, pelleted, and incubated in 2.3 M sucrose overnight at 4°C. Subsequently, cell pellets were mounted on silver pins, flash frozen and stored in liquid nitrogen. The specimens were sectioned with a Reichert Ultracut S ultramicrotome with a Reichert FCS cryo-attachment using a Diatome diamond knife (Diatome, Biel, Switzerland). Double-labeling of thawed cryo-sections was performed as described [54],[55]. Protein A-gold (Utrecht University, Utrecht, Netherlands) of different sizes was used to label different viral proteins. EM specimens were examined using a Zeiss EM10 transmission electron microscope. Quantification of the labeling for anti-core and anti-NS5A was done using three different labeling experiments considering two grids per experiments. The average labeling per lipid droplet was estimated by considering 20 profiles of lipid droplets per grids and by counting the labeling on the surrounding membrane of the LD only. Statistical analysis was done by t-test using the free p- value calculater for 158 degree of freedom (http://www.graphpad.com/quickcalcs/Pvalue2.cfm).

## Supporting Information

Protocol S1 Plasmid Construction and Northern Blot
(0.03 MB DOC)
