# Supplementary material for: Essential Role of Domain III of Nonstructural Protein 5A for Hepatitis C Virus Infectious Particle Assembly
Source: PLoS Pathog. 2008 Mar 28;4(3):e1000035. doi: 10.1371/journal.ppat.1000035 (PMC2268006; doi:10.1371/journal.ppat.1000035)
Supplement: Protocol S1 — Plasmid Construction and Northern Blot (0.03 MB DOC) [file ppat.1000035.s001.doc]

**SUPPLEMENTARY INFORMATION**

**Plasmid construction.** Plasmid pFK-Jc1/ΔE1E2 that carries a deletion of 350 codons in the E1-E2 coding region (removing amino acids 218 − 567 of the J6 polyprotein) was created by overlapp PCR. Two amplification cycles were performed by using primers carrying the new crossover sites and primers flanking the *Cla*I and *Hpa*I restriction sites (S/2A/343 5’ggaggtctcgtagaccgtgcaccatg3’; A/J6/E1E2 5’agggtggtgcctggagctgccaggtaatgc3’; S/A/J6/E1E2 5’gcagctccaggcaccaccctgccgtactag3’; A/2A/3089 5’cgtcagagctcacgctctgataag3’). The restricted and purified PCR fragment was then inserted into plasmid pFK-Jc1. NS5A deletion mutants were also generated by overlapp PCR by using the outside primers S6767 (5’cataggtttgcacccacaccaaag3’) and A-NS5B (5’ccatgtcatactcctggacc3’), or A-HpaI (5’gacttgatgtggttaacggccctc3’), or A7839 (5’gtcataatgggcgtcgagcacttg3’) and primers spanning the new crossover site. For generation of mutant Δ2222-2280 the overlapp PCR fragment was digested with *SanD*I and *Rsr*II and inserted into pFKi341PiLucNS3-3`JFH1. The fragment containg the deletion Δ2354-2435 was inserted into a *SanD*I and *Hpa*I digested pFKi341PiLucNS3-3`JFH1 replicon. To obtain Jc1-Luc genomes carrying deletions Δ2222-2280 or Δ2354-2435, *Mlu*I-*Spe*I fragments were transferred from the corresponding subgenomic replicons into pFKJc1-Luc. Plasmids pFKJc1-Luc-Δ2354-2404, -Δ2405-2435 and -Δ2328-2435 were generated by three-fragment ligation by using the *SanD*I/*BsrG*I digested PCR fragment and vector fragments obtained by restriction with *Mlu*I and *SanD*I or *BsrG*I and *Mlu*I. To obtain pFK-Jc1 genomes lacking the luciferase reporter gene and carrying the NS5A deletions Δ2222-2280, Δ2354-2404, Δ2405-2435 or Δ2354-2435, *SanD*I and *Mlu*I fragments were transfered from the corresponding pFK-Jc1-Luc plasmids into pFK-Jc1. Plasmid pFK-Jc1 carrying the Δ2328-2435 deletion was generated by overlap PCR as described above. To obtain pFK-Jc1/ΔE1E2+Δ2328-2435, a *Kpn*I/*Mlu*I fragment derived from the corresponding pFK-Jc1/Δ2328-2435 construct was inserted into the *Mlu*I/*Kpn*I digested pFK-Jc1/ΔE1E2 vector. For transcomplementation analysis we used plasmids pFKI389Luc-EI/NS3-3’_dg_JFH [1] and pFKI389Luc-EI/NS3-3’_dg_JFH/ Δ2328-2435. The latter was obtained by insertion of a *Nsi*I/*Hpa*I fragment spanning the deletion into pFKI389Luc-EI/NS3-3’_dg_JFH. All PCR-amplified sequences were confirmed by automated nucleotide sequencing with an ABI 310 sequencer (Applied Biosystems). Big Dye version 1.1 (Applied Biosystems) was used for cycle sequencing according to the manufacturer’s protocol.

**Northern Blot analysis.** Total RNA was prepared by a single-step isolation method [2]. After gel electrophoresis and blotting, the membrane was incubated with a [^32^P]-labeled negative-sense riboprobe complementary to nucleotides 4106 to 6007 of the JFH-1 genome as described previously [3]. Hybridization with a ß-actin-specific antisense riboprobe served to correct for the total RNA amount loaded in each lane of the gel. Signals were detected and quantified by autoradiography and phospho imaging.

**References of supplementary information**

1. Schaller T, Appel N, Koutsoudakis G, Kallis S, Lohmann V, et al. (2007) Analysis of hepatitis C virus superinfection exclusion by using novel fluorochrome gene-tagged viral genomes. J Virol 81: 4591-4603.

2. Chomczynski P, Sacchi N (1987) Single-step method of RNA isolation by acid guanidinium thiocyanate-phenol-chloroform extraction. Anal Biochem 162: 156-159.

3. Koutsoudakis G, Kaul A, Steinmann E, Kallis S, Lohmann V, et al. (2006) Characterization of the early steps of hepatitis C virus infection by using luciferase reporter viruses. J Virol 80: 5308-5320.
